# Comparison between Capsule Endoscopy and Magnetic Resonance Enterography for the Detection of Polyps of the Small Intestine in Patients with Familial Adenomatous Polyposis

**DOI:** 10.1155/2012/215028

**Published:** 2012-02-09

**Authors:** E. Akin, A. Demirezer Bolat, S. Buyukasik, O. Algin, E. Selvi, O. Ersoy

**Affiliations:** ^1^Department of Gastroenterology, Ankara Ataturk Research and Education Hospital, 06800 Ankara, Turkey; ^2^Department of Radiology, Ankara Ataturk Research and Education Hospital, 06800 Ankara, Turkey; ^3^Department of Gastroenterology, Yildirim Beyazit University, 06800 Ankara, Turkey

## Abstract

*Objective*. The objective of this study was to assess the utility of magnetic resonance enterography (MRE) compared with capsule endoscopy (CE) for the detection of small-bowel polyps in patients with familial adenomatous polyposis (FAP). *Methods*. Patients underwent MRE and CE. The polyps were classified according to size of polyp: <5 mm (small size), 5–10 mm (medium size), or >10 mm (large size). The location (jejunum or ileum) and the number of polyps (1–5, 6–20, >20) detected by CE were also assessed. MRE findings were compared with the results of CE. *Results*. Small-bowel polyps, were detected by CE in 4 of the 6 (66%) patients. Three patients had small-sized polyps and one patient had medium-sized polyps. CE detected polyps in four patients that, were not shown on MRE. Desmoid tumors were detected on anterior abdominal wall by MRE. *Conclusion*. In patients with FAP, CE can detect small-sized polyps in the small intestine not seen with MRE whereas MRE yields additional extraintestinal information.

## 1. Introduction

Familial adenomatous polyposis (FAP) is a disease with autosomal dominant inheritance. It is caused by an alteration of the FAP (APC) gene that is located on chromosome 5q21. The syndrome is characterized by the presence of adenomatous polyps in the gastrointestinal tract, mainly in the colon, rectum, and duodenum and is associated with 100% risk of colorectal cancer [1–3]. Patients with FAP have a cumulative lifetime risk of over 80% for developing duodenal adenomas, which are the precursor lesions for duodenal adenocarcinoma. Consequently, these patients have a 4% lifetime risk of periampullary or duodenal adenocarcinoma [4, 5]. In order to minimize the complications of small-bowel polyposis in FAP, upper endoscopy and small-bowel radiographic surveillance are recommended [6]. 

There is little doubt that capsule endoscopy (CE) is the best method to evaluate the entire small-bowel mucosa [7]. Studies have shown that CE is useful and safe surveillance of jejunal-ileal polyps in selected patients with FAP [8, 9]. However, capsule endoscopy has limitations including evidence that size estimation and anatomic localization of polyps at capsule endoscopy may be inaccurate even if experienced readers review the examinations [10]. Furthermore, polyps can be double-counted or missed altogether because of a combination of retrograde peristalsis, rapid capsule transit, and limited video frame capture rate [11–14]. CE is contraindicated in patients suspected of bowel stricture or obstruction [15]. 

Magnetic resonance enterography (MRE) has proven to be a reliable technique for the evaluation of mucosal abnormalities. MRE could be an ideal imaging modality to follow patients with FAP, being noninvasive, well tolerated, and radiation-free [16–18].

The aim of this study was to prospectively assess CE and MRE in the diagnostic work-up of patients with FAP.

## 2. Materials and Methods

Six patients with FAP were prospectively recruited between June 2010 and April 2011: five men and one woman (median age, 39.6 years; age range, 22–51 years), and examined by using CE and MRE. Patients excluded were those with severe swallowing disorders, claustrophobia, implanted cardiac pacemaker or other electronic devices, pregnant women, patients with a clinical suspicion of small-bowel obstruction/pseudoobstruction, strictures or fistulas, and children under 16 years old.

Experienced endoscopist (O. E.) reported all the videos. The polyps were classified into 3 groups: <5 mm, 5–10 mm, or >10 mm. The location (jejunum or ileum) and the number of polyps (1–5, 6–20, >20) detected by CE were also assessed. The location of small-bowel polyps was estimated by analyzing the CE transit time between pylorus passage and pouch-ileostomy (ileorectal anastomosis or ileocecal valve). The duodenum was designated to be the small bowel visualized up to 2 min after pylorus passage. The remaining CE transit time was divided into three thirds of which the upper two thirds were designated jejunum and the lower third was presumed to be ileum.

All patients gave their written informed consent for CE and MRE exams. The study was approved by the local ethical committees. The procedures were performed in the morning, after an overnight fast. Bowel preparation was performed with 4 L polyethylene glycol (PEG) solution given 15 hours before the procedure. CE was performed by using the PillCam SB with the RAPID workstation and software (Given Imaging Ltd, Yoqneam, Israel). Patients were allowed to drink fluids 2 hours after capsule ingestion and were allowed a light meal 4 hours later. Data were recorded for approximately 8 hours. After data sampling, the recorder was disconnected, and all data were downloaded to the workstation and analyzed on the following day.

### 2.1. MRE Technique

In all patients, MRE was also performed within 2 weeks after CE in separate occasions. Patients were asked to fast the night before the examination, and bowel enema was not given for intestinal preparation. 1500 mL oral contrast agent solution (containing 10 grams of methylcellulose, 200 mL PEG, and 1300 mL water) was prepared, homogenized, and administered for intestinal distention. Oral-contrast agent was ingested over a 30–45 min period, as permitted by patients' tolerance and cooperation. After drinking up the whole solution, 20 mg of hyoscine-*N*-butyl bromide (Buscopan, Boehringer Ingelheim, Germany) was given intravenously to suppress bowel spasm and motion, and then patients were taken to the MR suite. 

All MRE studies were performed by using a 1.5 Tesla MR machine (Philips Intera Achieva, Best, The Netherlands) by using a phased-array body coil in supine position. After acquiring three-plane scout images, two-dimensional (2D) turbo spin-echo (TSE) T2- weighted (W), 2D-TSE-T1W images and three-dimensional (3D) T1W (THRIVE) gradient-echo images were obtained. After intravenous administration of gadoterate-meglumine (0.1 mmol/kg; Dotarem, Guerbet, France), T1W sequences were repeated with same parameters in portal and delayed phases. MRE reported by one radiologist (O. A.). 

## 3. Results

Of the 6 FAP patients, 3 had previously undergone a proctocolectomy with ileoanal pouch anastomosis, 1 had a subtotal colectomy with ileorectal anastomosis, and 2 had no surgery. Patient demographics, prior operative history, and a summary of CE and MRE findings are outlined in Table 1. All patients successfully underwent MRE and CE. Complete passage of the small bowel by CE was obtained in all patients who performed CEs. No complications related to capsule endoscopy or MRE were observed in any of the study patients. Patients tolerated both methods. Image quality was satisfactory in all patients.

**Figure 1:**
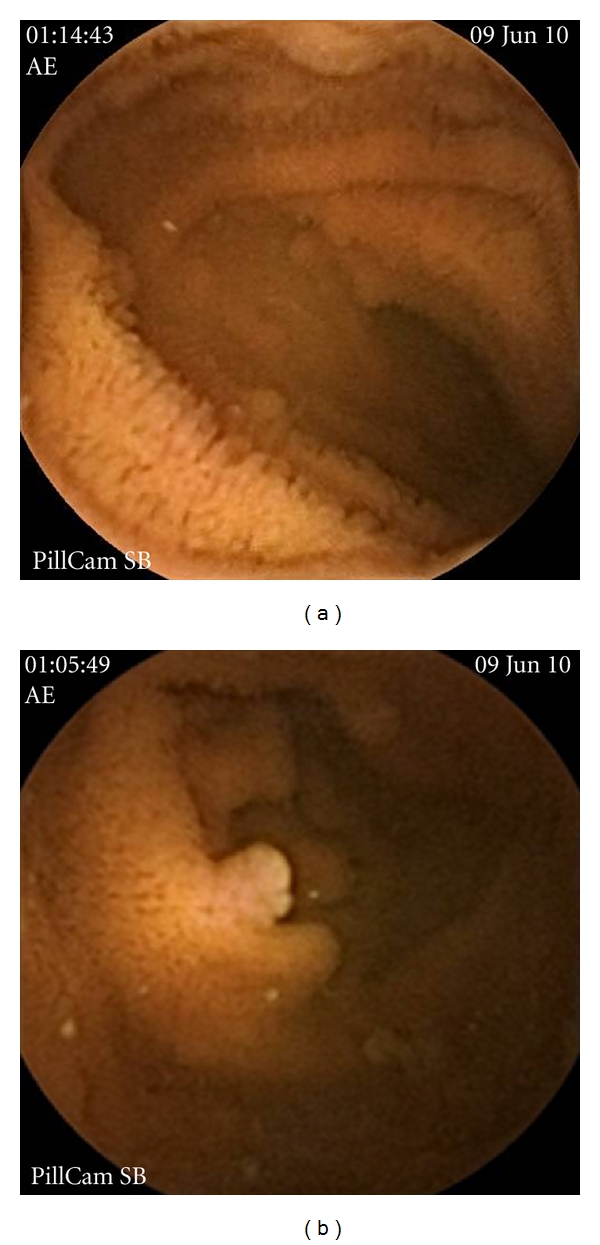
CE detected small-sized (<5) (a) and medium-sized (5–10 mm) (b) polyps.

**Figure 2:**
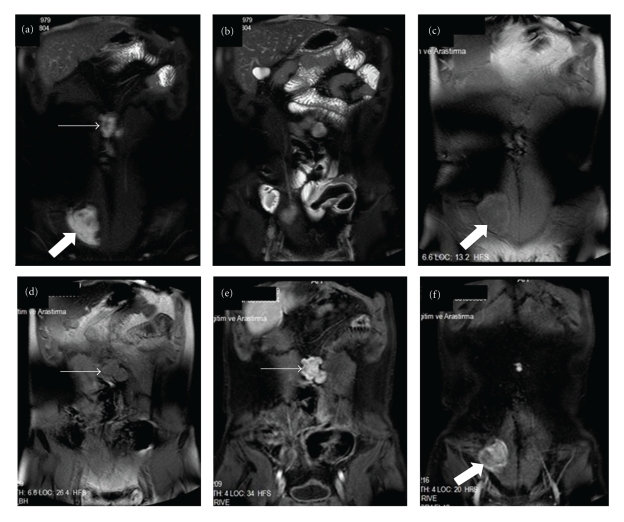
MR enterographic images of the patient 3. On coronal fat-saturated T2- weighted (W) HASTE (a), (b), T1W (c), (d), and contrast-material-enhanced T1W (e), (f), images show the desmoid tumors (thick arrows: inguinal desmoid tumor; thin arrows: mesenteric desmoid tumor). The relationship between contrast-material-enhanced mesenteric desmoid tumor and small-bowel loops is clearly seen on the MR images (arrow, (e)).

Small-bowel polyps were detected by CE in 4 of the 6 (66%) FAP patients ranging from estimated <5 mm to 5–10 mm in size (Table 1). Thereof, in three patients small-sized (<5) and in one patient, medium-sized (5–10 mm) polyps were seen (Figures 1(a) and 1(b)). CE detected polyps in four patients that were not shown on MRE. In one patient (number 3) MRE showed two desmoids tumors, one of them 62 × 59 mm in midabdominal area invading into the left rectus muscle and the other 43 × 38 mm extending from anterior abdominal wall to subcutaneous tissue (Figures 2(a)–2(f)). These tumors were excised.

## 4. Discussion

FAP is a hereditary polyposis syndrome with a high risk for benign small-bowel polyps and cancer. Hence, endoscopic surveillance of the upper gastrointestinal tract with forward-viewing and lateral-viewing endoscopy, as well as ileoscopy, is recommended [8, 19]. Upper gastrointestinal lateral-viewing endoscopy is highly effective for identifying most polyps within the duodenum. However the possibility of adenomas developing in segments of the bowel inaccessible by standard upper GI endoscopy in a proportion of FAP patients indicates that additional modes of screening could be considered. Small-intestinal adenomas prevalence in FAP patients varies, depending of the modality used for their detection [20].

CE and MRE have both emerged relatively recently and are increasingly utilized for small-bowel assessment. Technical advances have enhanced MRE's diagnostic capability in small-bowel imaging. CE is noninvasive, safe, and comfortable and can be performed on an ambulatory basis in FAP patients. Recently, CE has been shown to be effective for the detection of small-bowel polyps [8, 9].

A few previous studies already assessed the diagnostic value of MRE in the evaluation of patients with FAP, in comparison with capsule endoscopy, with preliminary satisfactory results [17, 18]. In a study investigating diagnostic value of MRE in the Peutz-Jeghers syndrome (PJS), this method showed 93% concordance with enteroscopy [21]. The authors concluded that MRE could be used for surveillance of PJS patients. Caspari et al. compared CE and MRE for detection of small-intestinal polyps. Polyps larger than 15 mm were equally detected by the two methods. However, smaller lesions were better shown by CE [17]. Tescher et al. also reached similar results in a study of 20 FAP cases [19].

In patients with FAP, CE can detect small-sized polyps in the small intestine not seen with MRE whereas MRE yields additional extraintestinal information. Probably, CE-detected polyps in our study were not seen by MRE as they were smaller than 15 mm. Small-intestinal air-fluid levels might have masked the polyps in MRE. Also, insufficient small-intestinal distention might have obscured them. CE may be more valuable in detection of small polyps as it shows the mucosa directly. Due to the small size of the detected polyps in this series, no histology was obtained and no polypectomy was performed. There is no sufficient data concerning clinical relevance of these small-intestinal polyps in FAP patients and necessity for surveillance yet. Further studies are warranted. Gupta et al. compared CE to MRE in 19 PJS patients. The two methods showed similar performance in detection of the polyps of 10 to 15 mm. However, MRE was more effective in recognition of those larger than 15 mm [18]. Such a comparison was not made in our study due to low patient number and as we did not observe polyps larger than 10 mm.

 One of the limiting features of CE is that it may not detect periampullary lesions well. In a study of patients undergoing CE for various reasons, Clarke et al. found 10.4% sensitivity of CE in showing the major papilla [22]. Duodenal polyps are usually adenomatous and have a 4–12% cancer risk [19]. Therefore, side vision endoscopy is recommended for visualization of periampullary region in FAP patients [22]. Studies investigating the value of MRE in imaging of the 2nd part of duodenum may be planned.

FAP is a multisystem disorder of growth. Affected individuals can develop thyroid and pancreatic cancer, hepatoblastomas, CNS tumors (especially medulloblastomas), and various benign tumors such as adrenal adenomas, osteomas, desmoid tumors, and dental abnormalities. Prophylactic colectomy has improved the life expectancy of patients, as a result of which the prevalence of other manifestations has increased [23]. In our study population, MRE showed desmoid, tumors in one patient, and they were surgically removed. MRE may be useful in detection of extraintestinal lesions in FAP patients. CE only gives the possibility of investigating intraluminal space and mucosa. On the other hand MRE is able to show all layers of the small intestine and it allows identification of extraluminal pathologies, too [24]. In this aspect, MRE may be more advantageous than CE in FAP patients.

In conclusion, both CE and MRE can be used for screening the small bowel in patients with FAP. CE is capable of detecting smaller polyps which can be missed by MRE. However, MRE is superior for detecting larger polyps with additional advantage of a rapid overview on mural, perienteric, and extraenteric information. There is no recent data concerning the clinical significance of detection of such small polyps. MRE might be the ideal modality for surveillance for this reason. Therefore, further studies are needed to clarify this matter.

## Figures and Tables

**Table 1:** Demographic, clinical, CE, and MRE findings of the patients.

No	Age	Sex	Prior surgery	CE polyps	Jejunum	Ileum	MRE
**1**	M	51	Subtotal colectomy + ileorectal anastomosis	6–20/<5 mm	+	+	−
**2**	M	22	No	>20/5–10 mm	+	+	−
**3**	M	31	Subtotal colectomy + ileorectal anastomosis	1–5/<5 mm	−	+	−
**4**	M	28	Subtotal colectomy + ileorectal anastomosis	0	−	−	−
**5**	F	26	No	0	−	−	−
**6**	M	44	Proctocolectomy − ileoanal pouch anastomosis	1/<5 mm	+		−

MRE: MR enterography, CE: capsule endoscopy, A: anastomosis.
